# Supplementary material for: Functional ultrasound neuroimaging reveals mesoscopic organization of saccades in the lateral intraparietal area
Source: Nat Commun. 2025 Oct 1;16:8752. doi: 10.1038/s41467-025-63826-z (PMC12488887; doi:10.1038/s41467-025-63826-z)
Supplement: Supplementary file 1 — Supplementary Information [file 41467_2025_63826_MOESM1_ESM.pdf]

Supplementary Figures

A

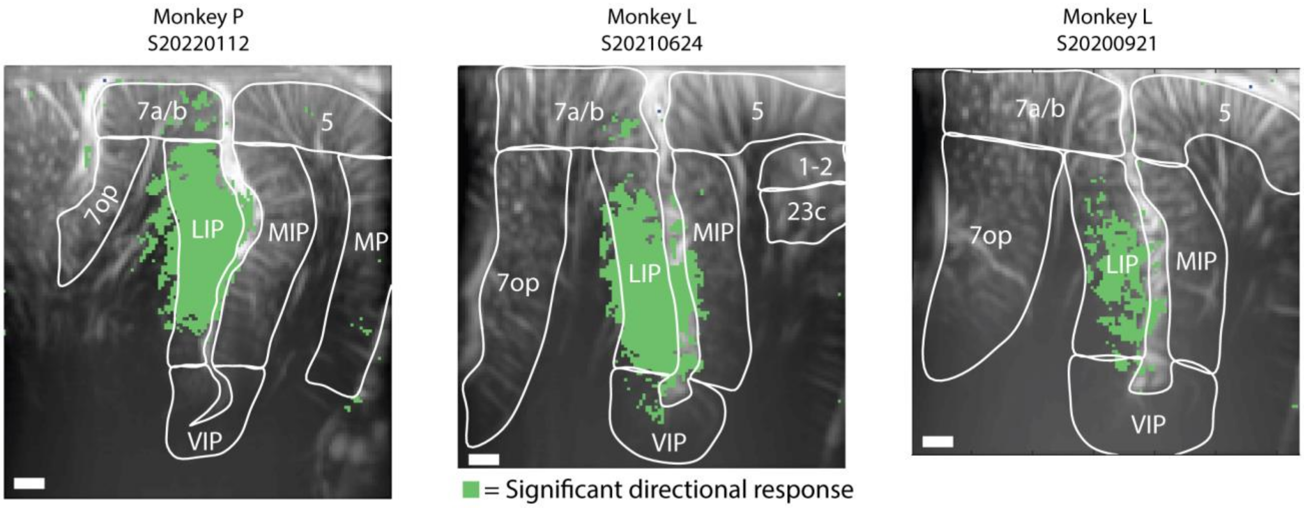

B

Percent of voxels with significant directional response

7op	0.0%	7op	0.1%	7op	0.0%
7a/b	8.5	7a/b	2.6	7a/b	0.0
LIP	80.1	LIP	71.2	LIP	33.9
VIP	0	VIP	5.3	VIP	2.0
MIP	1.6	MIP	7.1	MIP	0.3
5	0.3	5	0.0	5	0.1
MP	0.6	Area 1-2	0.0		
		Area 23c	0.0		

Fig. S1 – Non-LIP regions show minimal directional response
A. Statistical overlays of voxels with significant directional response (green voxels) during the last second of the memory period (single timepoint within +/-0.5 seconds of memory end). Anatomical labels and boundaries estimated based upon Saleem et al. 2012[26]. White scalebar = 1mm. **B.** Percent of voxels with significant directional response. Calculated as (voxels with significant directional response)/(number of voxels within anatomical region).

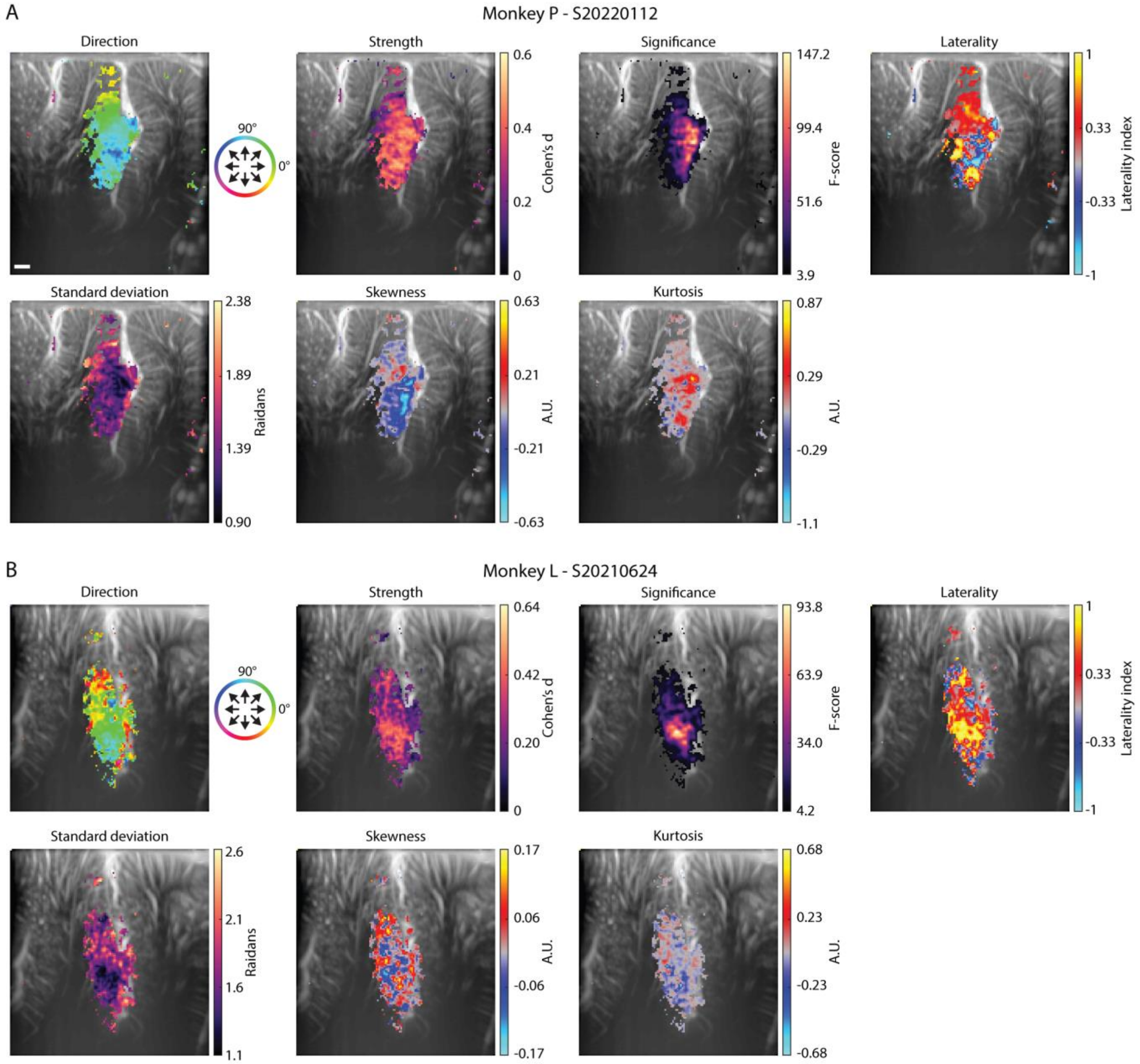

Fig. S2 – Supplement to Fig. 3; Voxel-wise statistical measures for example sessions
A. Statistical overlays for example session from Monkey P (S20220112). All statistical measures calculated at end of memory period. Directional preference calculated using the center-of-mass approach described in main paper methods. Strength plot shows length of center-of-mass vector. Significance plot shows voxel-wise F-score from GLM model. Standard deviation, skewness, and kurtosis calculated using `Circular Statistics Toolbox` in MATLAB[86]. White scalebar = 1mm. **B.** Statistical overlays for example session from Monkey L (S20210624). Same format as **(A)**.

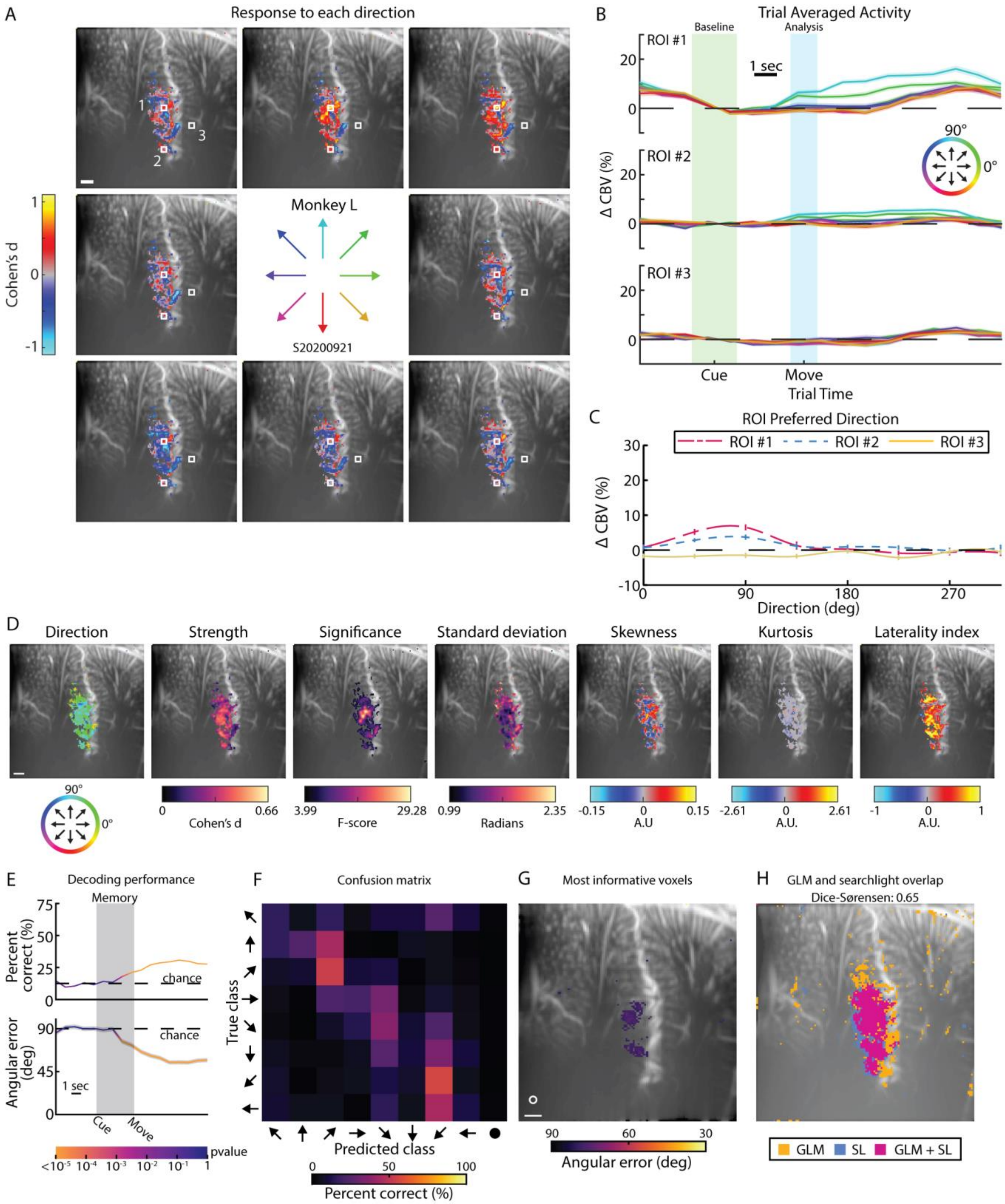

Fig. S3 – Second example session in Monkey L (S20200921)

A-C. GLM analysis. Same format as Fig. 3D-F. **D.** Statistical distribution metrics for voxel-wise tuning curves. Same format as Fig. S2. **E-G.** Decoding analysis. Same format as Fig. 4D-F. **H.** Overlap of GLM and searchlight analysis statistical masks. Overlap calculated using Dice-Sørenson metric. For all panels, plots based upon n=532 trials. Source data are provided as a Source Data file.

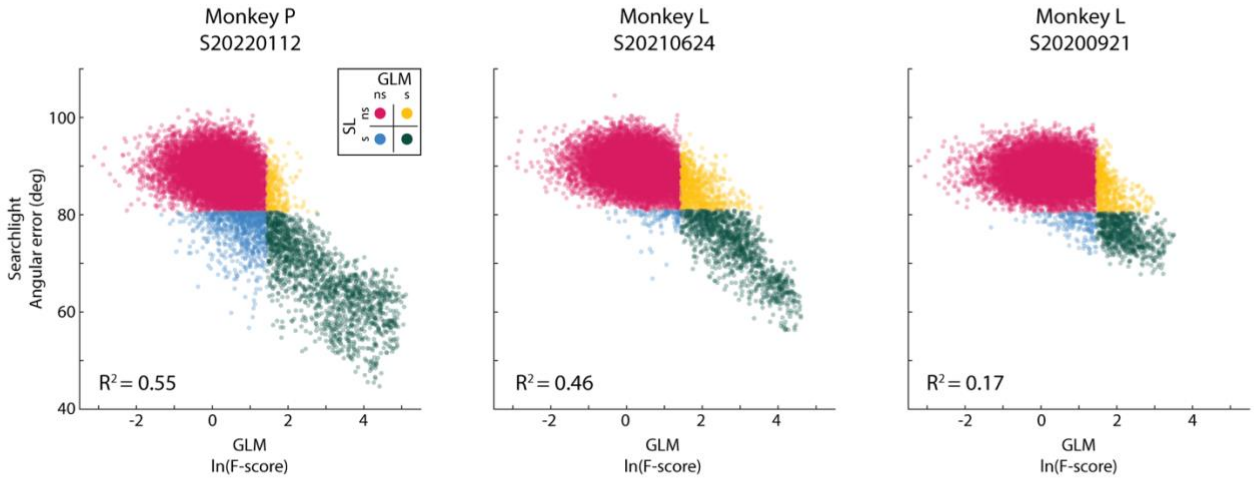

Fig. S4 – Correlation between GLM weight and searchlight accuracy for each example session
Voxel-wise relationship between GLM weights and searchlight accuracy. Different dot colors represent voxel-wise statistical significance for searchlight and/or GLM value (q<0.001). Source data are provided as a Source Data file.

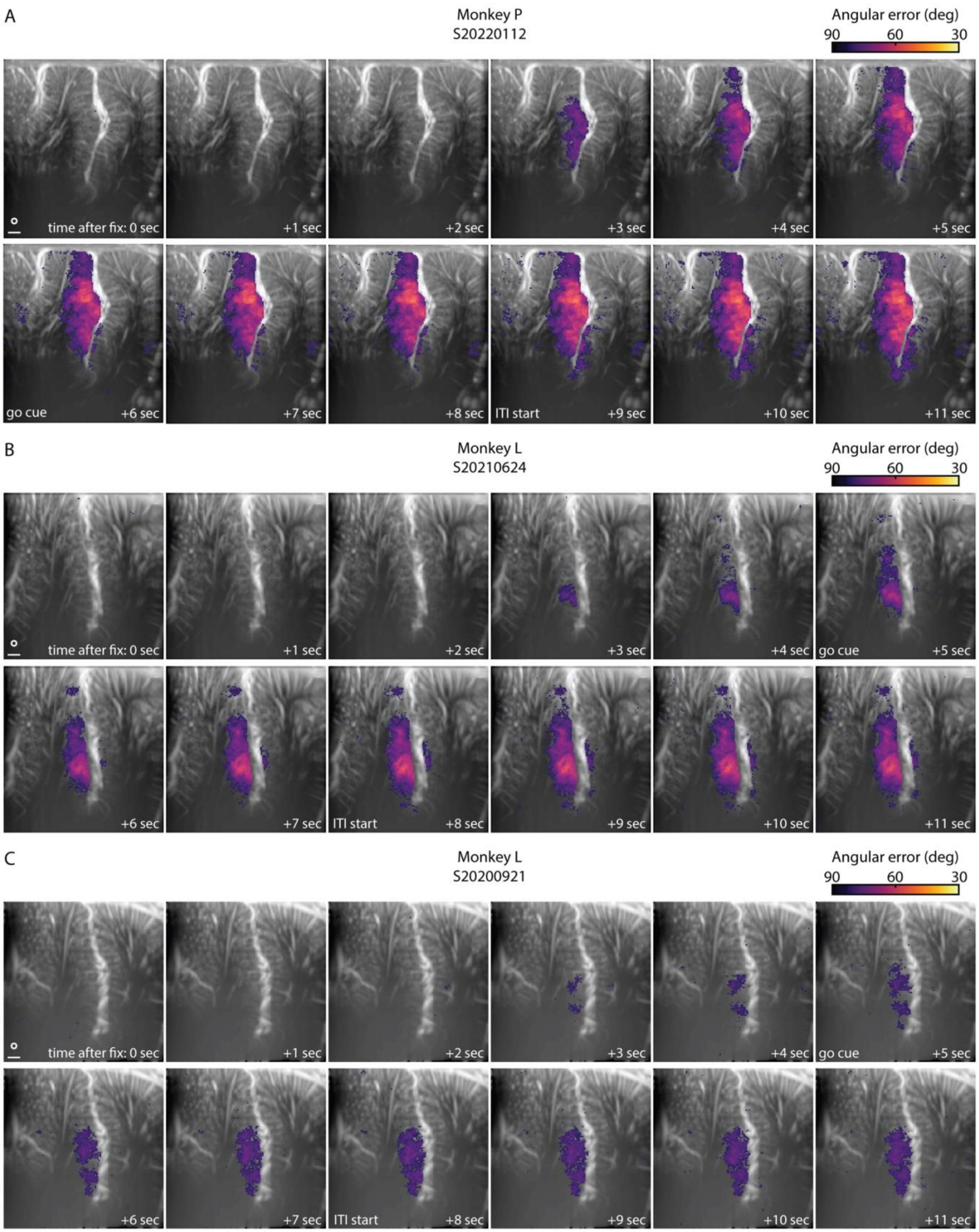

Fig. S5 – Supplement to Fig 4; Searchlight results across decoding time windows

A. Example session in Monkey P (S20220112). Each tile shows searchlight analysis for that timepoint. All significant decoding ($p<0.001$) shown. Per methods in main paper, at each timepoint after the cue, we used all previous timepoints after the cue in the trial. For example, to test our ability to decode at 2 seconds after cue onset, we concatenated the data from 0, 1, and 2 seconds after the cue. White circle – 200 µm searchlight radius. White line – 1 mm scalebar. **B.** Example session in Monkey L (S20210624). Same format as **(A)**. **C.** Example session in Monkey L (S20200921). Same format as **(A)**.

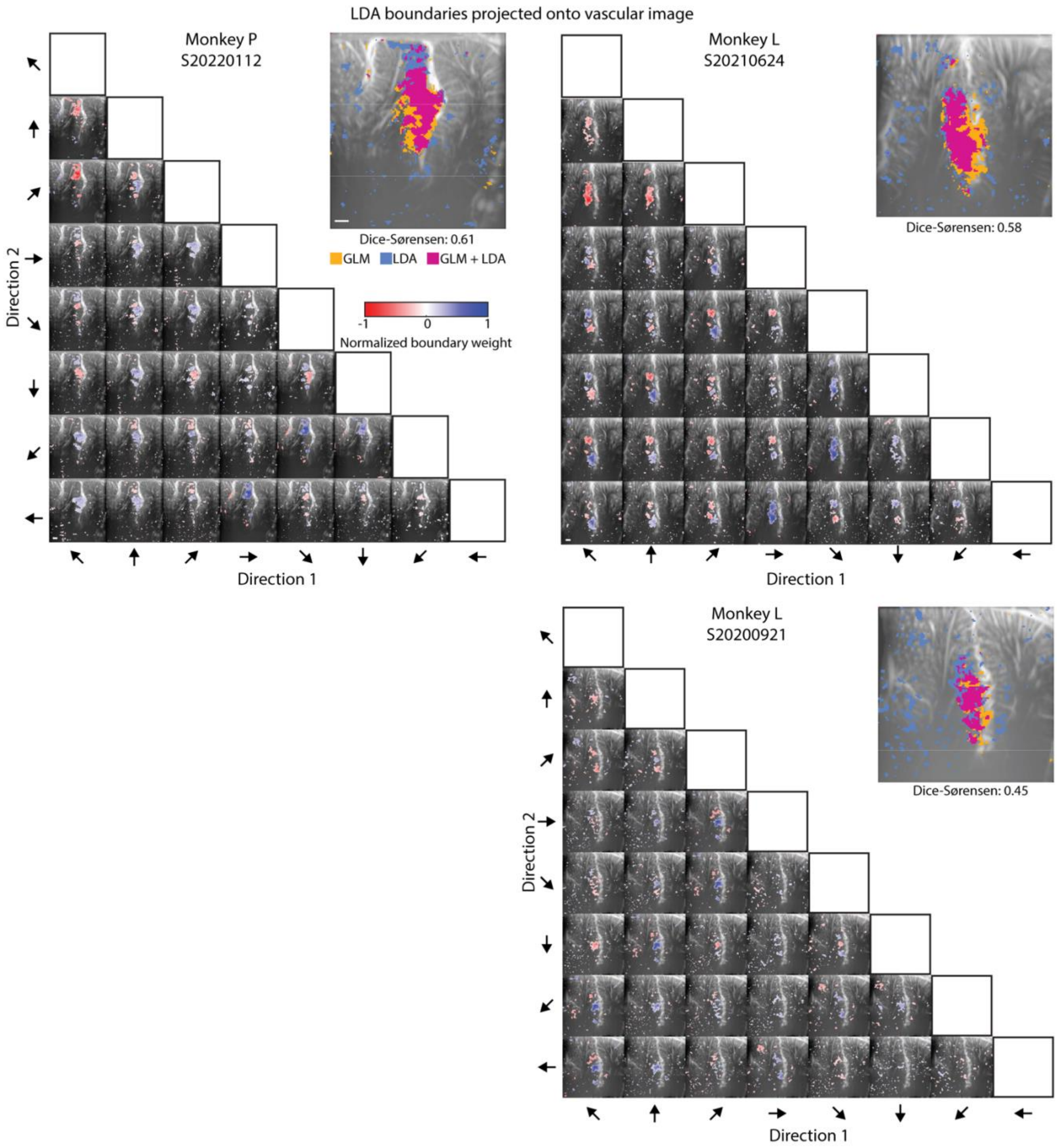

Fig. S6 – Projection of LDA weights onto vascular images for example sessions

For each session, each tile represents the LDA boundary between two direction classes. Each tile calculated by multiplying the voxel-wise horizontal and vertical LDA weights together after projection through the inverse PCA transformation. Insets show the overlap between GLM-identified voxels (q<1e-5, FDR-corrected) and the 10% of voxels with the strongest LDA weights across all directional pairs, quantified using Dice-Sørenson similarity.

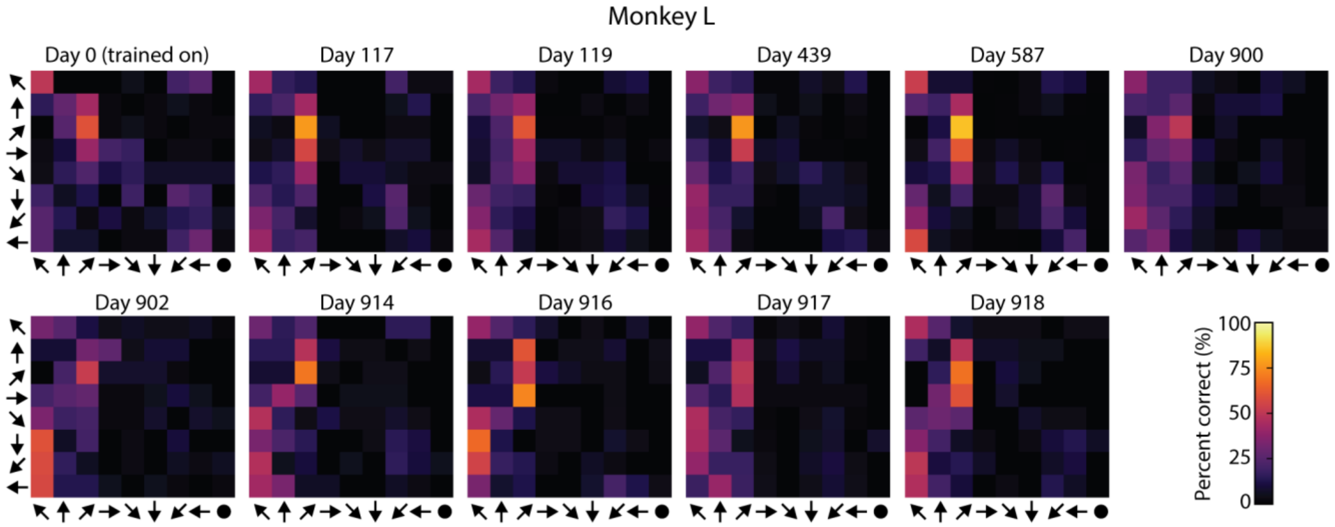

Fig. S7 – Stability of directional preference across time.
Same format as **Fig. 5D** but using a different training session. The decoder was trained on Day 0 data and tested on other sessions from the same imaging plane without any retraining. Source data are provided as a Source Data file.

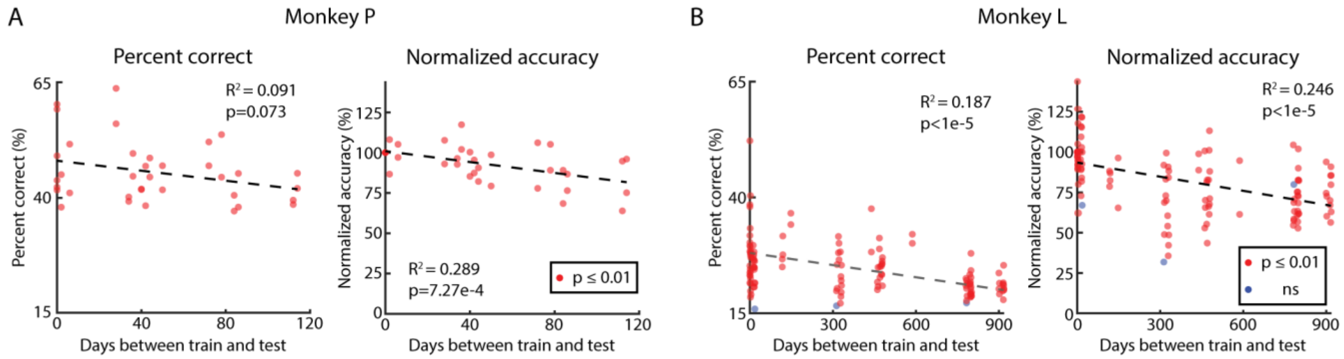

Fig. S8 – Supplement to Fig. 5; Pairwise accuracy as function of time between training and testing session
A. Absolute and normalized accuracy plots for Monkey P as a function of days between the training and testing session (absolute difference in time). Normalized accuracy is normalized to performance on 10-fold cross-validation performance on training set. Dashed lines – Linear fit to data. **B.** Absolute and normalized accuracy plots for Monkey L. Same format as **(A)**. Source data are provided as a Source Data file.

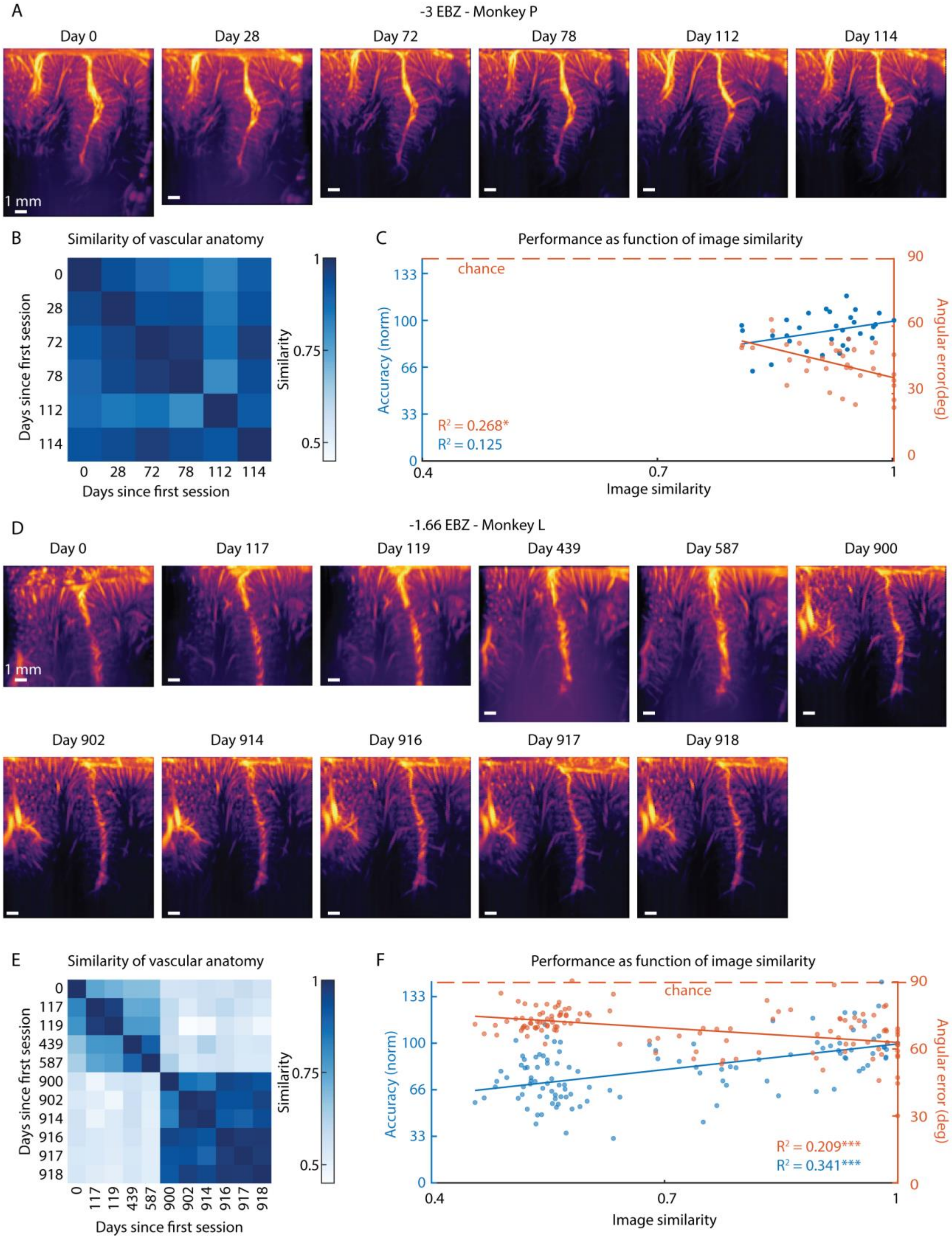

Fig. S9 – Impact of image similarity on decoder performance.
A. Vascular anatomy for each recording session from the same recording slot in Monkey P. White scalebar – 1 mm. **B.** Pair-wise similarity between different vascular images for Monkey P. **C.** Performance as a function of image similarity for Monkey P. Left axis (blue) shows normalized accuracy. Right axis (orange) shows mean angular error. Each session is represented by a pair of blue and orange dots. *=$p<10^{-2}$, **=$p<10^{-4}$, ***=$p<10^{-6}$. **D-F.** Same format as **(A-C)** for Monkey L. Source data are provided as a Source Data file.

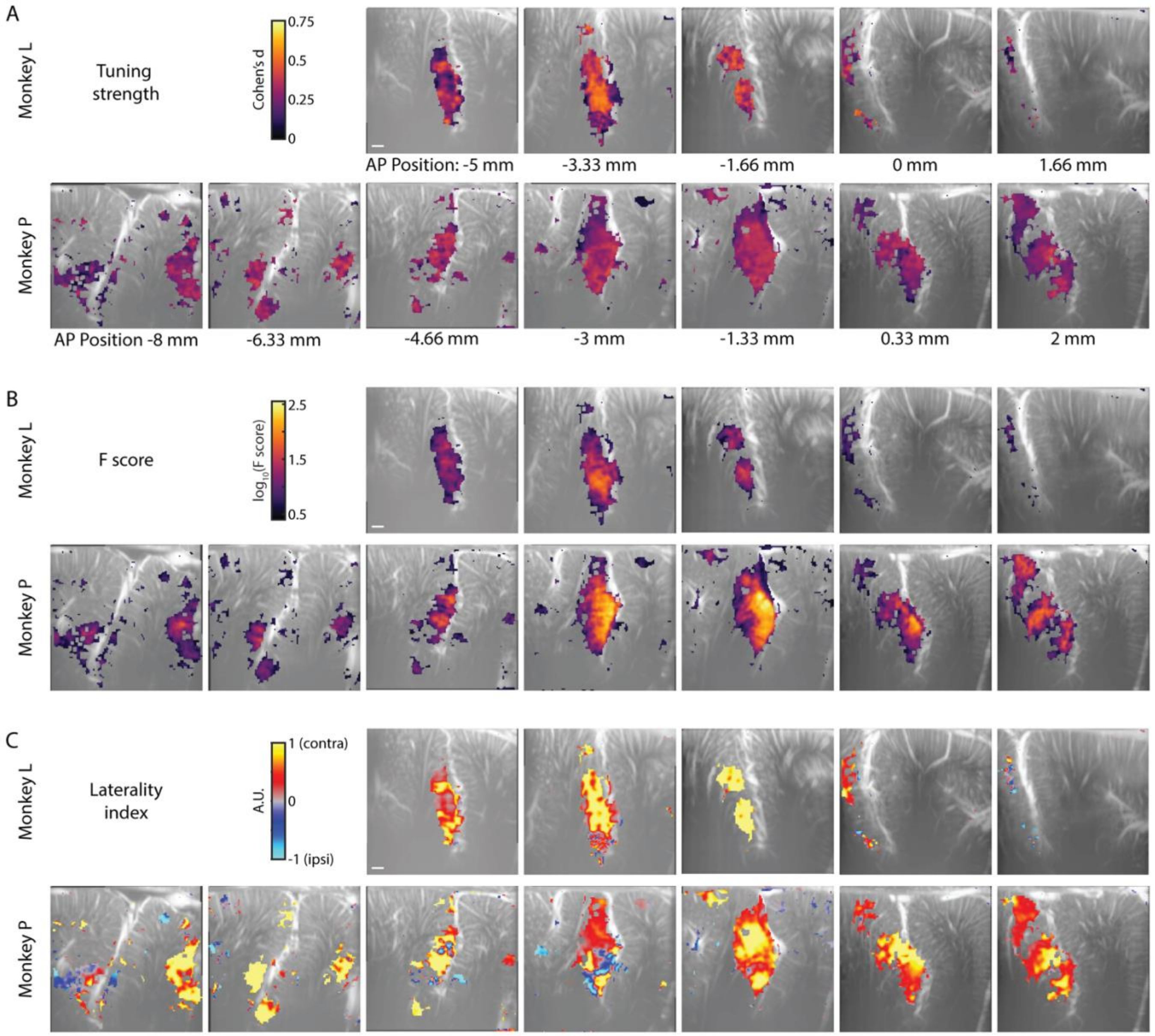

Fig. S10 – Supplement to Fig. 6; Voxel-wise statistical measures of strength for each coronal plane

A. Each tile shows voxel-wise strength value for center-of-mass analysis. White scalebar = 1mm. **B.** Each tile shows voxel-wise log_{10}(F-score) from GLM model for all sessions from that coronal plane. **C.** Each tile shows voxel-wise laterality index.

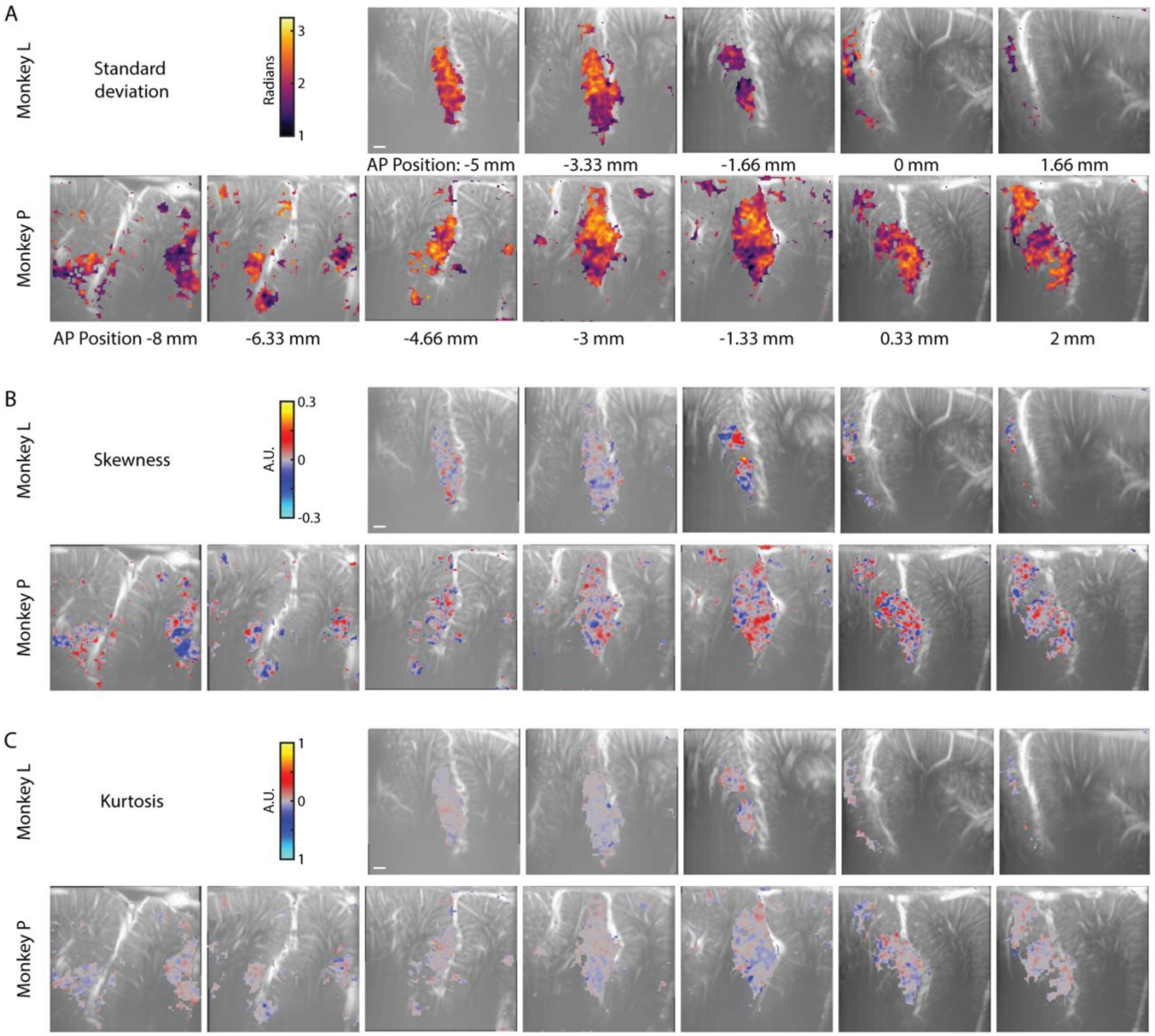

Fig. S11 – Supplement to Fig. Voxel-wise statistical measures of data distribution for each coronal plane
A. Each tile shows voxel-wise circular standard deviation calculated using Circular Statistics Toolbox. White scalebar = 1mm. **B.** Each tile shows voxel-wise angular skewness calculated using Circular Statistics Toolbox. **C.** Each tile shows voxel-wise angular kurtosis calculated using Circular Statistics Toolbox.

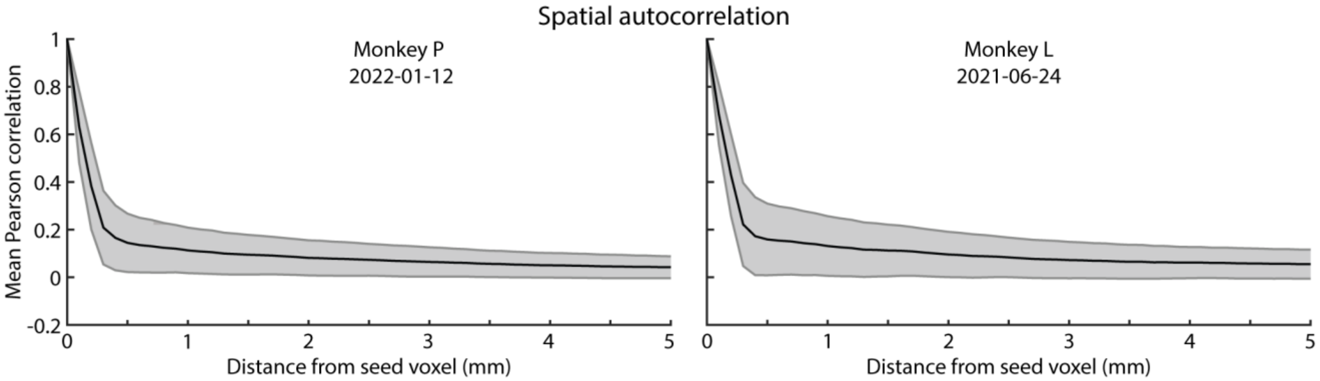

Fig. S12 – Power Doppler data displays spatial autocorrelation.
Mean Pearson spatial autocorrelation across entire fUSI field. Voxel radius – Distance of voxels from center seed voxel. Shaded area – standard deviation. Source data are provided as a Source Data file.

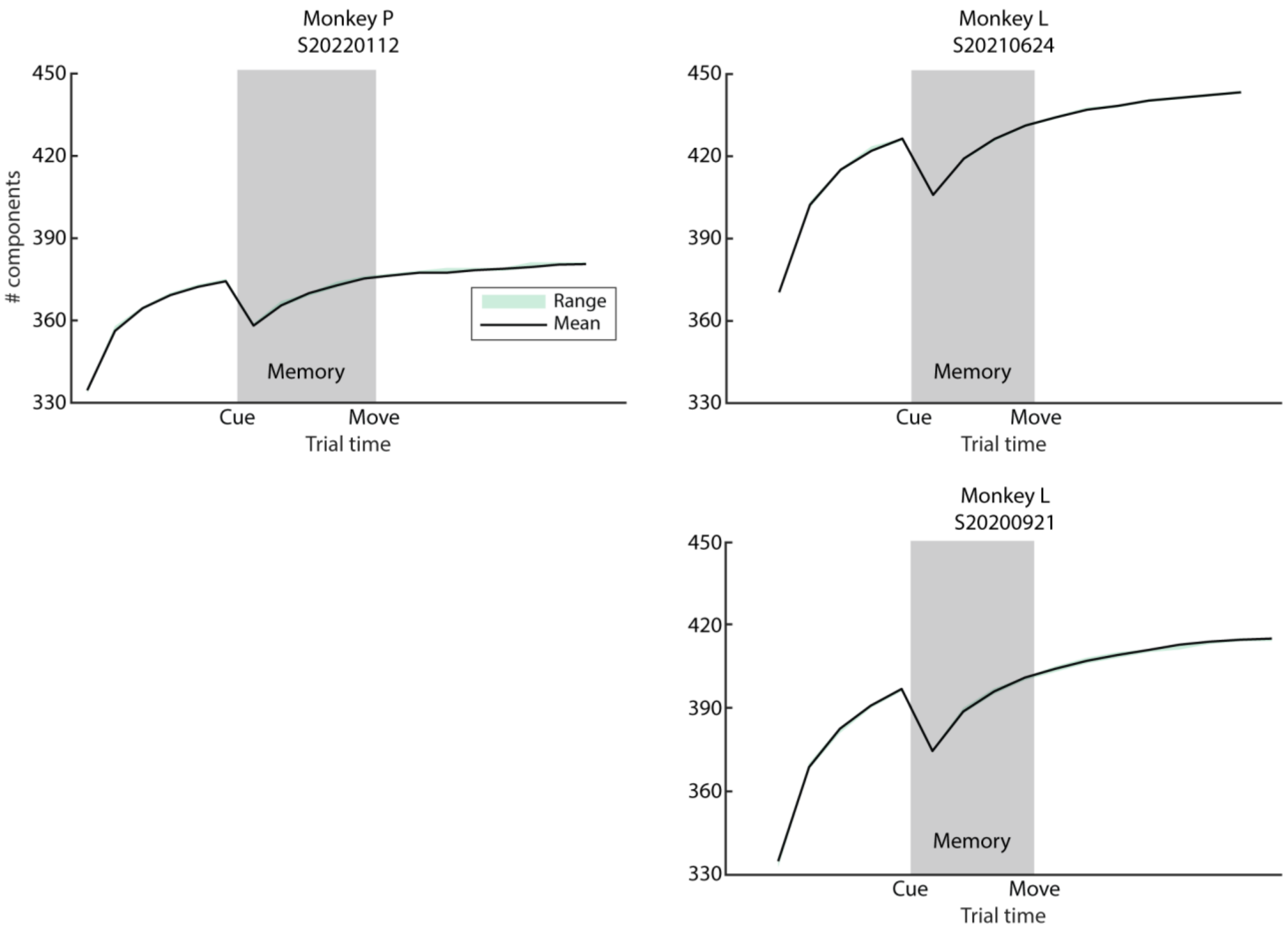

Fig. S13 – Number of components to capture 95% variance in Principal Component Analysis for each example session
For each example session, plot shows the number of components kept to capture 95% of the variance in the training data. Green region represents the range of components kept for each fold in a 10-fold cross-validation. Black shows mean components kept. Per methods in main paper, at each timepoint after the cue, we used all previous timepoints after the cue in the trial. For example, to test our ability to decode at 2 seconds after cue onset, we concatenated the data from 0, 1, and 2 seconds after the cue. We treated these timepoints as additional features in the decoder model. In other words, the input to our decoder model had N*T features, where N is the number of voxels in a single Power Doppler image and T is the number of timepoints. Source data are provided as a Source Data file.

Supplementary Table 1 – Metadata from fUSI sessions

Collection Date	Monkey	Coronal plane (Relative to EBZ)	Notes
2019-11-15	Monkey L	-3.33	
2020-03-11	Monkey L	-3.33	
2020-03-13	Monkey L	-3.33	
2020-07-07	Monkey L	-3.33	
2020-08-26	Monkey L	-1.66	
2020-08-28	Monkey L	-5	
2020-09-02	Monkey L	-5	
2020-09-16	Monkey L	-1.66	
2020-09-21	Monkey L	-5	Example session
2020-09-30	Monkey L	0	
2020-10-07	Monkey L	1.66	
2020-10-14	Monkey L	1.66	
2021-01-27	Monkey L	-3.33	
2021-03-05	Monkey L	0	
2021-03-12	Monkey L	0	
2021-03-19	Monkey L	0	
2021-03-22	Monkey L	0	
2021-03-26	Monkey L	1.66	
2021-06-11	Monkey L	-1.66	
2021-06-24	Monkey L	-3.33	Example session
2021-12-08	Monkey P	-4.66	
2021-12-15	Monkey P	-4.66	
2021-12-17	Monkey P	-8	
2022-01-05	Monkey P	-8	
2022-01-07	Monkey P	-6.33	
2022-01-12	Monkey P	-3	Example session
2022-01-13	Monkey P	-1.33	
2022-01-14	Monkey P	0.33	
2022-02-02	Monkey P	2	
2022-02-04	Monkey P	3.66	
2022-02-09	Monkey P	-3	
2022-02-11	Monkey P	-1.33	
2022-02-17	Monkey P	-4.66	
2022-02-18	Monkey P	-6.33	
2022-02-23	Monkey P	-4.66	
2022-03-02	Monkey P	0.33	
2022-03-08	Monkey P	2	
2022-03-09	Monkey P	-3.33	

Supplementary Table 2 – Additional sessions used for longitudinal decoding analysis

From previous paper (Griggs and Norman et al. *Nature Neuroscience* 2024).

Collection Date	Monkey	Coronal plane (Relative to EBZ)	Notes
2022-03-25	Monkey P	-3	
2022-03-31	Monkey P	-3	
2022-04-22	Monkey L	0	
2022-05-03	Monkey L	-3.33	
2022-05-04	Monkey P	-3	
2022-05-05	Monkey L	-3.33	
2022-05-06	Monkey P	-3	
2022-05-17	Monkey L	-3.33	
2022-05-19	Monkey L	-3.33	
2022-05-20	Monkey L	-3.33	
2022-05-21	Monkey L	-3.33	
