# Supplementary material for: Functional ultrasound neuroimaging reveals mesoscopic organization of saccades in the lateral intraparietal area
Source: Nat Commun. 2025 Oct 1;16:8752. doi: 10.1038/s41467-025-63826-z (PMC12488887; doi:10.1038/s41467-025-63826-z)
Supplement: Supplementary file 2 — Description of Additional Supplementary Files [file 41467_2025_63826_MOESM2_ESM.pdf]

Description of Additional Supplementary Files

File name: Supplementary Movie 1
Description: Anatomy in Monkey P – 3D reconstruction of vascular anatomy in Monkey P.
Related to Fig. 2

File name: Supplementary Movie 2
Description: Anatomy in Monkey L – 3D reconstruction of vascular anatomy in Monkey L.
Related to Fig. 2
