# Supplementary material for: Functional ultrasound neuroimaging reveals mesoscopic organization of saccades in the lateral intraparietal area
Source: Nat Commun. 2025 Oct 1;16:8752. doi: 10.1038/s41467-025-63826-z (PMC12488887; doi:10.1038/s41467-025-63826-z)
Supplement: Supplementary file 5 — Reporting Summary [file 41467_2025_63826_MOESM5_ESM.pdf]

nature portfolio

Corresponding author(s): Whitney Griggs and Richard Andersen

Last updated by author(s): Aug 3, 2025

Reporting Summary

Nature Portfolio wishes to improve the reproducibility of the work that we publish. This form provides structure for consistency and transparency in reporting. For further information on Nature Portfolio policies, see our Editorial Policies and the Editorial Policy Checklist.

Statistics

For all statistical analyses, confirm that the following items are present in the figure legend, table legend, main text, or Methods section.

n/a	Confirmed	
☐	☒	The exact sample size (*n*) for each experimental group/condition, given as a discrete number and unit of measurement
☐	☒	A statement on whether measurements were taken from distinct samples or whether the same sample was measured repeatedly
☐	☒	The statistical test(s) used AND whether they are one- or two-sided<br>*Only common tests should be described solely by name; describe more complex techniques in the Methods section.*
☒	☐	A description of all covariates tested
☐	☒	A description of any assumptions or corrections, such as tests of normality and adjustment for multiple comparisons
☐	☒	A full description of the statistical parameters including central tendency (e.g. means) or other basic estimates (e.g. regression coefficient) AND variation (e.g. standard deviation) or associated estimates of uncertainty (e.g. confidence intervals)
☐	☒	For null hypothesis testing, the test statistic (e.g. *F*, *t*, *r*) with confidence intervals, effect sizes, degrees of freedom and *P* value noted<br>*Give P values as exact values whenever suitable.*
☐	☒	For Bayesian analysis, information on the choice of priors and Markov chain Monte Carlo settings
☒	☐	For hierarchical and complex designs, identification of the appropriate level for tests and full reporting of outcomes
☐	☒	Estimates of effect sizes (e.g. Cohen's *d*, Pearson's *r*), indicating how they were calculated

Our web collection on statistics for biologists contains articles on many of the points above.

Software and code

Policy information about availability of computer code

Data collection	We used a programmable high-framerate ultrasound scanner (Vantage 256; Verasonics, Kirkland, WA) to drive the ultrasound transducer and collect pulse echo radiofrequency data. We used a pulse repetition frequency of 7500 Hz with 5 evenly spaced tilted angles (-6° to 6°) with 3 accumulations to create one high-contrast compounded ultrasound image. We acquired the high-contrast compound images at 500 Hz and saved the images for offline construction of Power Doppler images. We constructed each Power Doppler image using 250 compound images acquired over 0.5 seconds.<br><br>For behavioral data tracking and collection, we used custom Python 2.7 software based on PsychoPy (v1.90.3). For eyetracking, we used an commercially available EyeLink 1000 system (Ottawa, Canada).
Data analysis	We used MATLAB 2021b and 2024b for all analyses. Code used to generate key figures and results is available at https://github.com/wsgriggs2/PPC_directional_tuning and archived on Zenodo at https://doi.org/10.5281/zenodo.15122174.

For manuscripts utilizing custom algorithms or software that are central to the research but not yet described in published literature, software must be made available to editors and reviewers. We strongly encourage code deposition in a community repository (e.g. GitHub). See the Nature Portfolio guidelines for submitting code & software for further information.

Data

Policy information about availability of data

All manuscripts must include a data availability statement. This statement should provide the following information, where applicable:

- Accession codes, unique identifiers, or web links for publicly available datasets
- A description of any restrictions on data availability
- For clinical datasets or third party data, please ensure that the statement adheres to our policy

Key data used in this paper is archived with CaltechDATA at https://doi.org/10.22002/p5jan-02r60

Research involving human participants, their data, or biological material

Policy information about studies with human participants or human data. See also policy information about sex, gender (identity/presentation), and sexual orientation and race, ethnicity and racism.

Reporting on sex and gender	N/A
Reporting on race, ethnicity, or other socially relevant groupings	N/A
Population characteristics	N/A
Recruitment	N/A
Ethics oversight	N/A

Note that full information on the approval of the study protocol must also be provided in the manuscript.

Field-specific reporting

Please select the one below that is the best fit for your research. If you are not sure, read the appropriate sections before making your selection.

☒ Life sciences ☐ Behavioural & social sciences ☐ Ecological, evolutionary & environmental sciences

For a reference copy of the document with all sections, see nature.com/documents/nr-reporting-summary-flat.pdf

Life sciences study design

All studies must disclose on these points even when the disclosure is negative.

Sample size	We determined the sample size (e.g., number of trials per experimental condition, number of sessions) based upon our lab's experience and standards in the field. This was consistent with our previous studies using a similar motor task (Norman et al. 2021 and Griggs and Norman et al. 2023).
Data exclusions	No data were excluded from the analysis.
Replication	We replicated our results in two rhesus monkeys, as widely practiced in the primate research field. We repeated the same task setup for multiple sessions per monkey.
Randomization	Randomization of animals to different groups was not relevant for our study. We randomly varied trial parameters, such as length of intertrial interval, to prevent the animals from anticipating task changes, which was important for studying movement planning in our study.
Blinding	Blinding was not relevant for our study because the trial parameters, such as movement direction, were randomly adjusted for each trial.

Reporting for specific materials, systems and methods

We require information from authors about some types of materials, experimental systems and methods used in many studies. Here, indicate whether each material, system or method listed is relevant to your study. If you are not sure if a list item applies to your research, read the appropriate section before selecting a response.

Materials & experimental systems

n/a	Involved in the study
☒	☐ Antibodies
☒	☐ Eukaryotic cell lines
☒	☐ Palaeontology and archaeology
☐	☒ Animals and other organisms
☒	☐ Clinical data
☒	☐ Dual use research of concern
☒	☐ Plants

Methods

n/a	Involved in the study
☒	☐ ChIP-seq
☒	☐ Flow cytometry
☒	☐ MRI-based neuroimaging

Animals and other research organisms

Policy information about studies involving animals; ARRIVE guidelines recommended for reporting animal research, and Sex and Gender in Research

Laboratory animals	We worked with two healthy 14-year-old male rhesus macaque monkeys (Macaca mulatta) weighing 14-17 kg.
Wild animals	No wild animal were used in this study.
Reporting on sex	Both rhesus macaque monkeys were male.
Field-collected samples	No field-collected samples were used in this study.
Ethics oversight	All training, recording, surgical, and animal care procedures were approved by the California Institute of Technology Institutional Animal Care and Use Committee and complied with the Public Health Service Policy on the Humane Care and Use of Laboratory Animals.

Note that full information on the approval of the study protocol must also be provided in the manuscript.

Plants

Seed stocks	*Report on the source of all seed stocks or other plant material used. If applicable, state the seed stock centre and catalogue number. If plant specimens were collected from the field, describe the collection location, date and sampling procedures.*
Novel plant genotypes	*Describe the methods by which all novel plant genotypes were produced. This includes those generated by transgenic approaches, gene editing, chemical/radiation-based mutagenesis and hybridization. For transgenic lines, describe the transformation method, the number of independent lines analyzed and the generation upon which experiments were performed. For gene-edited lines, describe the editor used, the endogenous sequence targeted for editing, the targeting guide RNA sequence (if applicable) and how the editor was applied.*
Authentication	*Describe any authentication procedures for each seed stock used or novel genotype generated. Describe any experiments used to assess the effect of a mutation and, where applicable, how potential secondary effects (e.g. second site T-DNA insertions, mosiacism, off-target gene editing) were examined.*
